# An Active Giant Cell Tumor of the Patella: A Case Report

**DOI:** 10.7759/cureus.1642

**Published:** 2017-09-02

**Authors:** Svetoslav A Slavchev, Georgi P Georgiev, Kircho Patrikov

**Affiliations:** 1 Department of Orthopaedics and Traumatology, Medical University of Sofia, Bulgaria, University Hospital of Orthopaedics - Sofia; 2 Department of Orthopaedics and Traumatology, Medical University of Sofia, Bulgaria, University Hospital Queen Giovanna

**Keywords:** giant cell tumor of bone, patella, knee pain, synthetic bone substitute, tricalcium phosphate, bone graft

## Abstract

Primary neoplasms of the patella account for less than 1% of all primary bone tumors of the lower extremity, the most frequent of them being the giant cell tumor of bone, the chondroblastoma, and the aneurysmal bone cyst. Herein, we report the case of a 29-year-old woman with an active giant cell tumor of the patella (GCTP) with its clinical and radiological features and a brief review of the literature.

## Introduction

A giant cell tumor of the bone (GCTB) is considered to be histologically benign, but it tends to be locally invasive and to form pulmonary metastases. With a prevalence of 4-5% of all primary bone lesions, these tumors develop in the metaphysis and epiphysis of the long bones and are staged as latent, active, or aggressive depending on the containment of the tumor within the bone, thinning of the cortex, or breaching of the bone by the tumor [1]. Although GCTB is usually located around the knee joint, patellar involvement is unusual. Its occurrence within this bone is under 1% of all GCTBs [2]. The most common symptoms are pain and/or swelling [1].

Here, we report our experience of its treatment through intralesional curettage, followed by bone grafting with a synthetic bone substitute, and make a brief literature review.

## Case presentation

A 29-year-old woman presented to our institution with anterior knee pain of two months duration. She had no history of strenuous activities or trauma. The pain was episodic at first, aggravated by prolonged walking, and later became constant and non-responding to NSAIDs.

**Figure 1:**
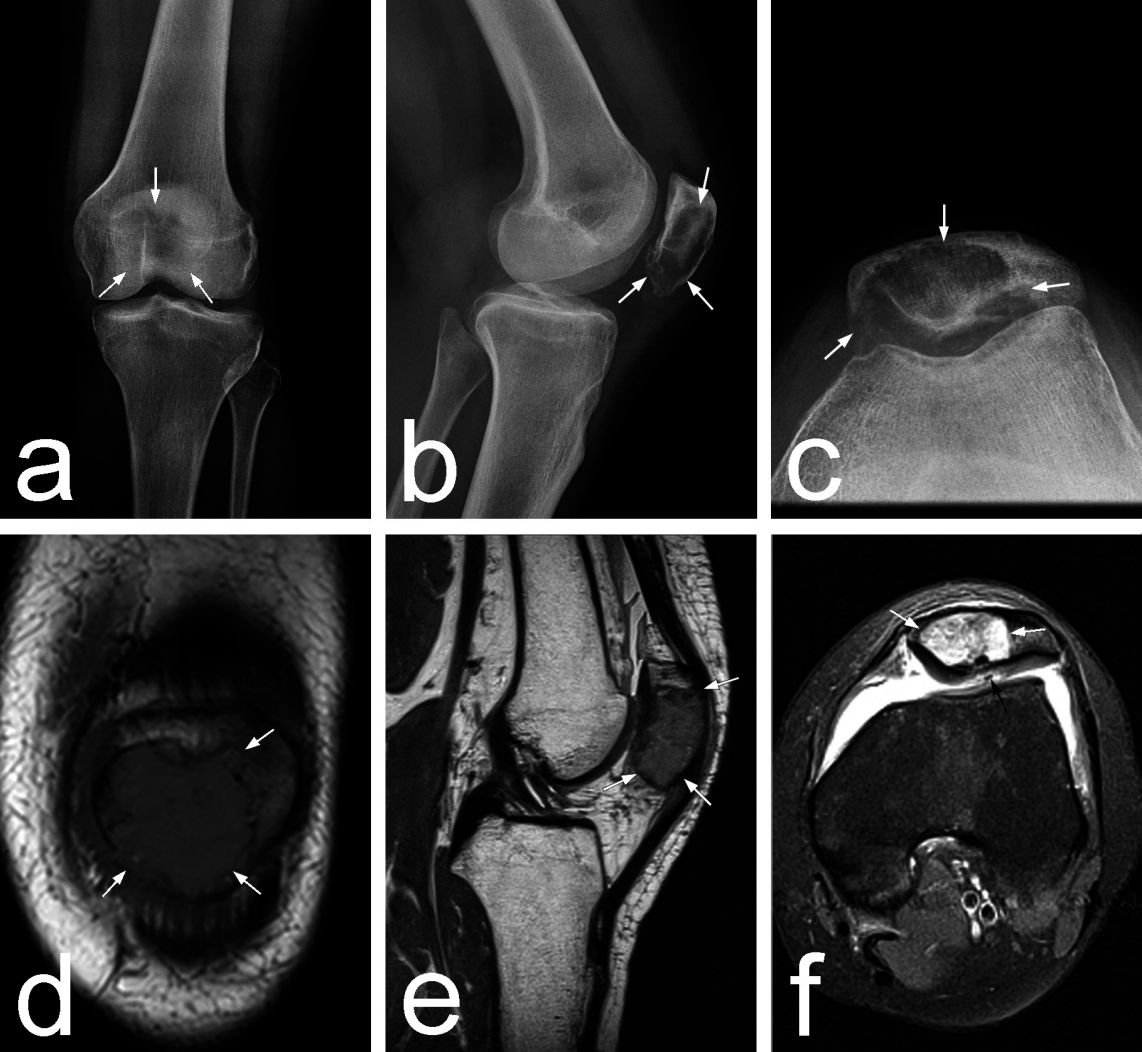
Plain radiographs and MRI at presentation 1A-1C: AP, lateral, and axial radiographs of the knee showing tumor extent (arrows). 1D-1F: Frontal, sagittal, and axial MRI slices trough the patella showing tumor extent (white arrows) and cartilage lesion (black arrow). MRI: magnetic resonance imaging

Physical examination revealed mild edema and tenderness over the patella and hypotrophy of the quadriceps. There was mild joint effusion, no skin changes, and no limitation of the range of motion. Radiography revealed a circumscribed septate osteolytic area in the distal three-quarters of the patella reaching the subchondral bone with endosteal scalloping and thinning of the cortical layer and remodeling of the subchondral bone with no periosteal reaction (Figures 1A-1C). Magnetic resonance imaging (MRI) showed a heterogeneous lesion contained within the bone with surrounding soft tissue edema and focal thinning of the patellar cartilage (Figures 1D-1F). An active GCTB was suspected, with chondroblastoma and aneurysmal bone cyst in the differential diagnosis.

**Figure 2:**
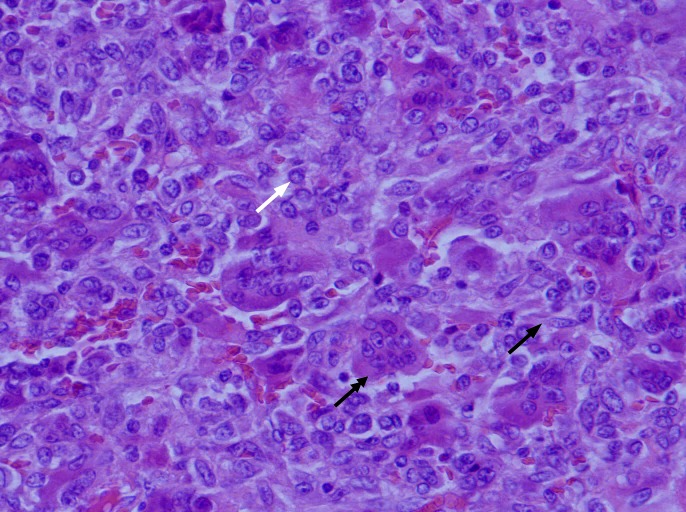
Hematoxylin and eosin stain of GCTB (x40). Round mononuclear cells (white arrow), spindle-shaped cells (black arrow), and multinucleated osteoclast-like giant cells (double arrow) in GCTB. GCTB: giant cell tumor of the bone

An open biopsy was performed, and GCTB was diagnosed with round and spindle-shaped mononuclear cells and abundant osteoclast-like giant cells (Figure 2).

**Figure 3:**
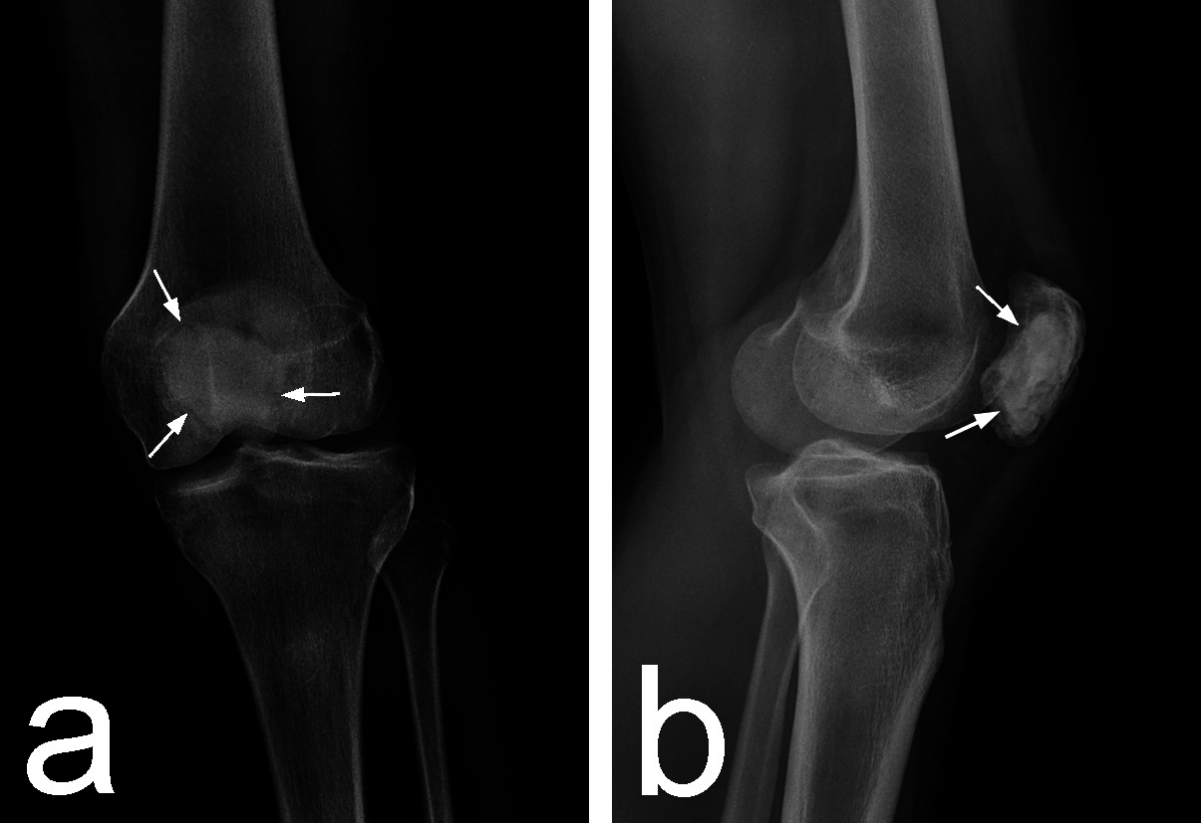
Plain radiographs at eight month follow-up A) AP and B) lateral radiographs of the knee showing "blurring" of the bone-TCP interface (arrows) as a sign of osteointegration of the graft. TCP: tricalcium phosphate

At the second stage, the patella was approached ventrally, 1 cm lateral to its medial margin. The lesion was thoroughly curetted through a wide window, allowing for direct visualization of its entire internal surface. Special attention was paid to the preservation of the subchondral bone, which was found to be deformed but not destroyed by the tumor. The main portion of the tumor tissue was a solid, yellowish-brown with multiple hemorrhages. The cavity was additionally curetted with a high-speed burr and filled with injectable tricalcium phosphate (TCP) bone cement. The wound was closed in the usual manner. Rehabilitation was initiated on the second postoperative day with a return to full function as tolerated towards the end of the fourth week. Eight months after surgery, there is radiographic evidence of osseointegration of the graft with no signs of local recurrence (Figures 3A-B) and the patient enjoys an active lifestyle with no pain or other troubles with her knee.

## Discussion

The patella is an unusual location for any primary or secondary bone tumor, and a neoplasm would rarely be considered in the etiology of anterior knee pain. Therefore, the diagnosis of a tumor within this location is delayed in most patients [3]. Benign lesions are more common and represent 70% of the tumors affecting the patella. The most common is the GCTB, followed by chondroblastoma, and aneurysmal bone cyst [4]. Other lesions that might affect the patella include metastases, lymphoma, Paget’s disease, osteosarcoma, chondrosarcoma, osteomyelitis, gout, brown tumor, osteoma, or solitary bone cyst [5].

According to a study of 27,403 primary bone tumors by the Bone and Soft Tissue Tumor Committee of the Japanese Orthopaedic Association spanning the period from 1972 to 2003, 13,860 of the tumors involved the bones of the lower extremity. Of those, 75 (0.5%) involved the patella, 71 (94%) being benign, GCTB accounting for 22 (31%) of cases, i.e., the GCTBs were 0.08% of all tumors of the patella and 0.15% of the bone tumors of the lower extremities [6]. Campanacci also reported that less than 1% of GCTBs arise in the patella [2]. Balke, et al. [7] reported on 214 cases of GCTB of which only two cases (0.9%) involved the patella. Singh, et al. [5] in 2009 presented 11 cases of GCTB out of 59 primary bone lesions of the patella.

The radiographic appearance of the GCTB of the patella is similar to GCTBs in other localizations. It typically presents as a lucent lesion without matrix calcifications growing often, but not exclusively, eccentrically in the meta-epiphyseal region of the bone, generally in a skeletally mature patient. In indolent and static tumors, the margins of the lesion are well-defined without a sclerotic rim. In aggressive cases, margins are poorly demarcated and the cortex may be thinned, distended, or destroyed with soft tissue extension, but a periosteal reaction is generally lacking. Complete or incomplete pathological fracture after bone destruction might also be detected [8].

As with other musculoskeletal neoplasms, computed tomography (CT) and MRI are essential in the evaluation and staging of the GCTB. CT is useful in the evaluation of the cortical bone and could clearly present thinning of the cortex, pathologic fracture, a periosteal reaction, and absence of matrix mineralization. In cases of cortical destruction, CT is surpassed by MRI’s ability to delineate the soft-tissue extension of the tumor where it appears with a heterogeneous signal that is low in T1-weighted images and high in T2-weighted images [1].

The surgical treatment includes curettage with bone grafting with or without local adjuvants, wide resection with patellar allografting, or patellectomy. Patellectomy is the preferred treatment for aggressive lesions [9]. In our case, despite the involvement of a very large portion of the patella, removal of the patella was not contemplated as a primary procedure because of the unimpaired overall function of the knee. Instead, aggressive curettage was performed, augmented by high-speed burring. TCP bone cement was chosen as the grafting material because of its mechanical strength in providing support for the subchondral bone, an absence of donor-site morbidity, and general radiographic homogeneity allowing for early detection of a local recurrence. Eventual replacement of TCP by viable bone tissue would provide a normal functioning patella. Packing the cavity with polymethyl methacrylate bone cement was considered at one time for its thermal adjuvant effect; however, it was dismissed because of the risk of thermal damage to virtually the whole articular surface of the patella that might eventually necessitate patellectomy in an otherwise healthy knee.

## Conclusions

Patellar tumors, despite being rare, should always be borne in mind in cases of anterior knee pain of uncertain origin. GCTB, followed by chondroblastoma and aneurysmal bone cysts, are the most common patellar tumors. The tumor stage must be taken into consideration when discussing treatment options. Besides the curative effect, the surgical treatment of non-malignant patellar tumors should strive for providing mechanical support for the subchondral bone and for ensuring enough viable bone in the long-term for withstanding the high mechanical loads of the knee.
